
JOURNAL INFORMATION
==============================
NLM Title Abbreviation: Ann N Y Acad Sci
Journal ID: Ann. N. Y. Acad. Sci
NLM Journal ID: 7506858
Journal ID: nyas

Annals of the New York Academy of Sciences

ISSN: 0077-8923
EISSN: 1749-6632

ARTICLE INFORMATION
==============================
PMCID: PMC3712481
DOI: 10.1111/nyas.12119
Article version: 1
Subjects: Issue Information

The Year in Diabetes and Obesity

Print publication date: 2013 Apr
Electronic publication date: 2013 Apr 1
Volume: 1281
Issue: 1
First page: i
Last page: i
Copyright: © 2013 The New York Academy of Sciences
Copyright year: 2013
License: Re-use of this article is permitted in accordance with the Creative Commons Deed, Attribution 2.5, which does not permit commercial exploitation.
License URL: https://creativecommons.org/licenses/by/2.5/

==============================
ANNALS *of* THE NEW YORK ACADEMY OF SCIENCES

VOLUME 1281

ISSUE

### The Year in Diabetes and Obesity

ISSUE EDITORS

Alvin C. Powers[a] and Rexford S. Ahima[b]

Vanderbilt University[a] and University of Pennsylvania[b]

EDITORIAL ADVISORY BOARD

**Diabetes**

**Mark Atkinson**
University of Florida

**Alan Attie**
University of Wisconsin–Madison

**Marshall Chin**
University of Chicago

**David D'Alessio**
University of Cincinnati

**Timothy Garvey**
University of Alabama at Birmingham

**David Harlan**
National Institute of Diabetes and Digestive and Kidney Diseases

**Steven Kahn**
University of Washington

**Christopher Newgard**
Duke University

**Jeffrey Pessin**
Albert Einstein College of Medicine

**Louis Philipson**
University of Chicago

**Camilo Ricordi**
University of Miami

**Elizabeth Seaquist**
University of Minnesota

**Gerald Shulman**
Yale University

**Obesity**

**Sheila Collins**
The Hamner Institutes for Health Sciences

**Roger Cone**
Vanderbilt University

**Michael Cowley**
Monash University, Australia

**Joel Elmquist**
University of Texas Southwestern Medical Center

**Sadaf Farooqi**
University of Cambridge

**Kevin Grove**
Oregon Health & Science University

**Samuel Klein**
Washington University in St. Louis

**Rudolf Leibel**
Columbia University

**Eleftheria Maratos-Flier**
Harvard Medical School

**Yuji Matsuzawa**
Osaka University

**Daniel Rader**
University of Pennsylvania

**Philipp Scherer**
University of Texas Southwestern Medical Center

**Randy Seeley**
University of Cincinnati

**Thomas Wadden**
University of Pennsylvania

The New York Academy of Sciences

Published by Blackwell Publishing
On behalf of the New York Academy of Sciences

Boston, Massachusetts
2013
